# Variable Assembly and Procapsid Binding of Bacteriophage P22 Terminase Subunits in Solution

**DOI:** 10.3390/pathogens13121066

**Published:** 2024-12-03

**Authors:** Julia Elise Cabral, Yanfei Qiu, Albert J. R. Heck, Reginald McNulty

**Affiliations:** 1 Laboratory of Macromolecular Structure, Department of Molecular Biology and Biochemistry, University of California Irvine, Steinhaus Hall, Irvine, CA 92697-3900, USA; jecabral@uci.edu (J.E.C.);; 2 Biomolecular Mass Spectrometry and Proteomics, Bijvoet Centre for Biomolecular Research and Utrecht Institute for Pharmaceutical Sciences, University of Utrecht, Netherlands Proteomics Center, Padualaan 8, 3584 CH Utrecht, The Netherlands; 3 Department of Pharmaceutical Sciences, University of California Irvine, Steinhaus Hall, Irvine, CA 92697-3900, USA

**Keywords:** *Salmonella* virus, viral genome-packaging motor, small terminase, large terminase, bacteriophage P22, electron microscopy

## Abstract

Concatemeric viral DNA is packaged into bacteriophage P22 procapsids via a headful packaging mechanism mediated by a molecular machine consisting of small (gp3) and large (gp2) terminase subunits. Although a negative stain reconstruction exists for the terminase holoenzyme, it is not clear how this complex binds the dodecameric portal protein located at a 5-fold mismatch vertex. Herein, we describe new assemblies for the holoenzyme. Both native mass spectrometry and transmission electron microscopy reveal that the P22 terminase complex adopts three main assemblies, which include a nonameric S-terminase bound to two L-terminase 1(gp3)_9_:2(gp2), two nonameric S-terminase bound to five L-terminase 2(gp3)_9_:5(gp2), and three nonameric S-terminase bound to seven L-terminase 3(gp3)_9_:7(gp2). Native agarose gel electrophoresis shows that the terminase complex interacts with procapsids with mild crosslinking. These results herein illustrate the P22 terminase complex can adopt a variety of conformations and assembly states.

## 1. Introduction

The intricate mechanisms of large dsDNA viruses, which include tailed bacteriophages, herpesviruses, poxviruses, and adenoviruses, have been extensively studied due to their impact on health and disease. For example, Herpes simplex virus-2 (HSV-2) affects approximately 45 million people and is a leading cause of genital herpes, with significant health disparities noted among different ethnic groups, particularly African Americans [1,2,3]. Furthermore, the intersection of HSV-2 with HIV highlights the critical interplay between viral infections and broader public health risks [1].

Parallel to the concerns presented by viral pathogens in humans are the challenges posed by bacterial infections, such as those caused by *Salmonella*. The lipopolysaccharide (LPS) membrane component of this pathogen is known to increase inflammation [4,5]. This bacterium is responsible for over 1.35 million illnesses and hundreds of deaths annually in the United States, with the potential for antibiotic-resistant strains to exacerbate this issue [6]. Given that *Salmonella* infections can arise from animal sources, interventions that target these bacteria at the zoonotic level are of paramount importance.

In this context, bacteriophage P22, a known predator of *Salmonella*, emerges as a biological entity of considerable interest. Understanding the genome packaging mechanism of bacteriophage P22 is not merely a pursuit of basic virology but has significant implications for public health. The packaging process is critical for phage replication and determines the infectivity and efficacy of the phage against its bacterial host. Insights into the molecular details of this process can inform the development of phage-based therapeutics, which hold promise as an alternative to traditional antibiotics, especially in the era of rising antimicrobial resistance.

Evidence of this potential is already seen with the P22 tailspike protein, which, when administered orally, has been shown to reduce *Salmonella* colonization in chickens [7]. By furthering our understanding of P22’s genome packaging, we may unlock new strategies to control and prevent *Salmonella* infections, potentially mitigating the public health burden associated with this pathogen.

**Figure 1:**
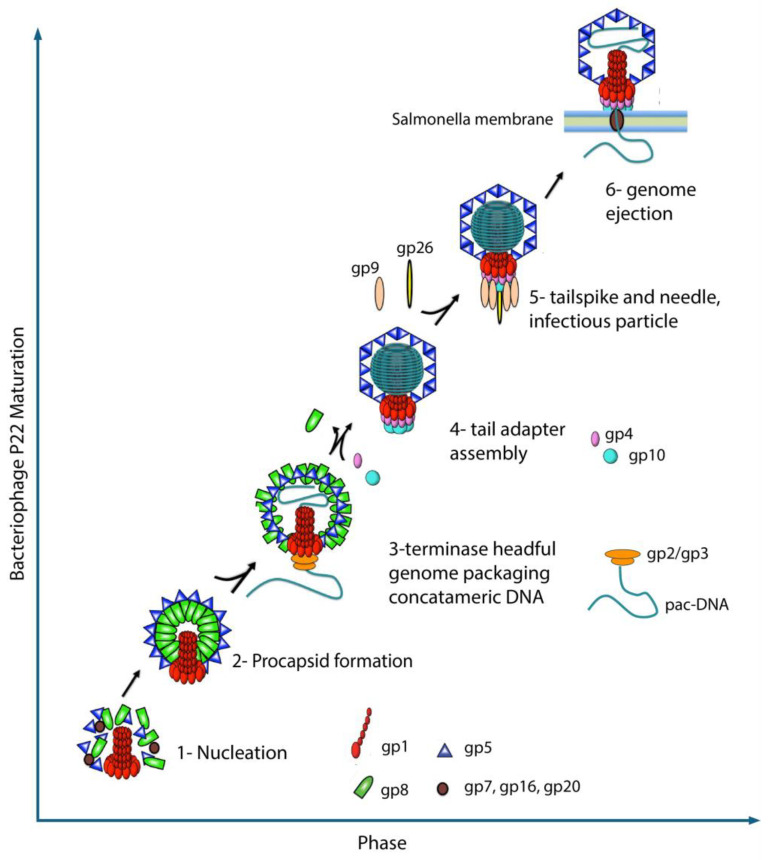
Schematic of bacteriophage P22 maturation. (1) Nucleation involves the portal protein (gp1), scaffold protein (gp8), injection proteins (gp7, gp16, and gp20), and coat protein (gp5). (2) A spherical procapsid forms with a dodecameric portal protein located at a symmetry-mismatched 5-fold vertex. (3) The large (gp2) and small (gp3) terminase complex binds to the pac site on P22 DNA. The terminase complex utilizes ATPase activity to package concatemeric DNA inside the procapsid, causing rearrangement of the coat proteins. Completion of headful packaging results in DNA cleavage via the gp2 nuclease domain. (4) Tail adapter proteins gp4 and gp10 bind to the portal vertex. (5) Tailspike (gp9) and needle protein (gp26) form the P22 tail. (6) The mature infectious particle is now capable of binding to the *Salmonella* membrane. Injection proteins (gp7, gp16, and gp20) form a tube, facilitating the safe delivery of the P22 genome into *Salmonella*.

The *Salmonella*-phage P22 packages its genome from concatemeric dsDNA using a powerful molecular motor that consists of large (gp2, 499 amino acids, 57.6 kDa [8]) and small (gp3, 162 amino acids, 18.6 kDa [9]) terminase subunits. In vitro studies have been performed that show that gp2 and gp3 form an oligomeric complex [10]. The recognition subunit, gp3, binds to packaging initiation (*pac*) sites [11] in the P22 genome and positions viral dsDNA to the large terminase subunit gp2, which uses ATP hydrolysis to translocate a single genome copy into empty procapsids. This reaction is very efficient and, in similar phages, proceeds at rates as high as 2000 bp/sec [12]. The nuclease domain of gp2 also cleaves the concatemeric dsDNA once the head is filled [11]. Upon cleavage, the gp3:gp2 complex quickly disassociates from the capsid, enabling binding of the tail factors gp4, gp10, and gp26 [13,14] that seal the portal protein and stabilize the genome inside the capsid. This is followed by the attachment of six copies of the trimeric tailspike gp9 [15] (Figure 1).

Gp3 spontaneously assembles into nine radially positioned protomers [16]. Initially, an 18 Å resolution structure obtained by negative stain electron microscopy (EM) revealed a C-terminally cleaved gp3 nonamer [17]. Subsequently, a crystal structure of 1.75 Å resolution along with the full-length EM structure of 18 Å resolution revealed an outer diameter of 95 Å, and an inner diameter of 23 Å, which is wide enough to accommodate hydrated dsDNA [2].

Gp2 consists of an N-terminal ATPase fold (residues 38–284), a flexible linker, a C-terminal nuclease domain (313–482), and a C-terminal basic tail (residues 483–499) [18,19,20]. The ATPase domain is conserved in phages and large-terminase-containing viruses. It contains a typical nucleotide-binding fold [21] with two subdomains containing ATP/GTP-binding Walker A and B motifs. The nuclease domain of gp2 has been solved via X-ray crystallography [22]. It has an RNAse H-fold and is a member of the ribonuclease H/resolvase/integrase superfamily [23]. The nuclease domain of gp2 has a mixed α/β fold and is globular in structure. The catalytic site has two Mg^2+^ ions. One Mg^2+^ is octahedrally coordinated with four Asp and four waters, while the other Mg^2+^ is tetrahedrally coordinated with Asn, His, and two waters. Catalytic site Asp^321^ is conserved in related phages and critical to nuclease activity [22].

The structure of the gp2 α-helical hairpin, residues 1–33, bound to Fab4 has been determined through crystallography [24]; however, the 3D structure of the P22 gp2 ATPase domain has not been determined to date. Since the fold is conserved, it is likely that the 3D structure resembles that of Sf6 [25] and T4 [26]. Monomeric gp2 assembles into a pentamer bound to the dodecameric portal (gp1) [27,28]. Bacteriophage P22 has a symmetry mismatch at one of its 5-fold vertices. This vertex contains the 12-fold portal protein, through which genome packaging and ejection occur.

There is a full-length crystal structure for the *S*f6 large terminase. The sequence identity of P22 to Sf6 is 15% and 12% for the corresponding large and small terminase, respectively [29]. Although the sequence identity is low for the large terminase, the 3D homology remains relatively high. Vis à vis other large terminases, we expect the 3D structure of monomeric gp2 to resemble that of other published large terminases.

A negative stain transmission electron microscopy (TEM) reconstruction has been determined for the first P22 terminase holoenzyme complex [30], which illustrates a stoichiometry of 1(gp3):2(gp2). Not only has a high-resolution cryo-EM structure not been determined since the negative stain reconstruction was reported but the stoichiometry of the complex of the large terminase complex that associates with the dodecameric portal has not been determined. The large terminase from T4 has been shown to form a pentamer and associate with the portal [31]. In this paper, we use a combination of electron microscopy coupled to mass spectrometry to show that the terminase complex can adopt variable stoichiometric assemblies, which include five copies of gp2. These results provide new insights into the biological relevance of heterogeneity observed in this dynamic molecular machine.

## 2. Materials and Methods

### 2.1. Biochemical Techniques

Full-length S-terminase (pMAL-S-terminase) and L-terminase (pET30b-L-terminase) were co-expressed in *E. coli* strain BL21-AI as previously described [2,22,32]. The S:L-terminase complex was expressed in *E. coli* strain BL21-AI (Life Technologies, Carlsbad, CA, USA) by inducing at 18 °C for 12–16 h with a final concentration of 0.2% L-arabinose and 0.1 mM IPTG. Cell pellets were resuspended in lysis buffer containing 20 mM Tris-Cl pH 8.0, 300 mM NaCl, 1 mM MgCl_2_, 3 mM β-mercaptoethanol, and 1 mM phenylmethylsulfonyl fluoride, and cells were disrupted by sonication. This prep differed from the 2015 prep in that it lacked glycerol [30]. The S:L-terminase complex was purified on amylose beads (New England Biolabs, Ipswich, MA, USA), and after washing with 500 mL of lysis buffer, the complex was incubated with 1 mM AMP-PNP (Sigma, St. Louis, MO, USA) and PreScission Protease to cleave off MBP. The cleaved species that came off the beads was further purified on a Superdex 200 16/60 gel filtration column (GE Healthcare, Chicago, IL, USA) in GF-buffer (20 mM Tris-Cl pH 8.0, 250 mM NaCl, and 1 mM MgCl_2_). The gel filtration column was calibrated with molecular weight markers as previously described [33]. Isolated S:L-terminase complex was concentrated to ~10 mg/mL using a 30 kDa MWCO ultrafiltration spin column (Vivaspin 20, Sartorius Stedim Biotech GmbH, Bohemia, NY, USA).

#### 2.1.1. EM Specimen Prep

**Figure 2:**
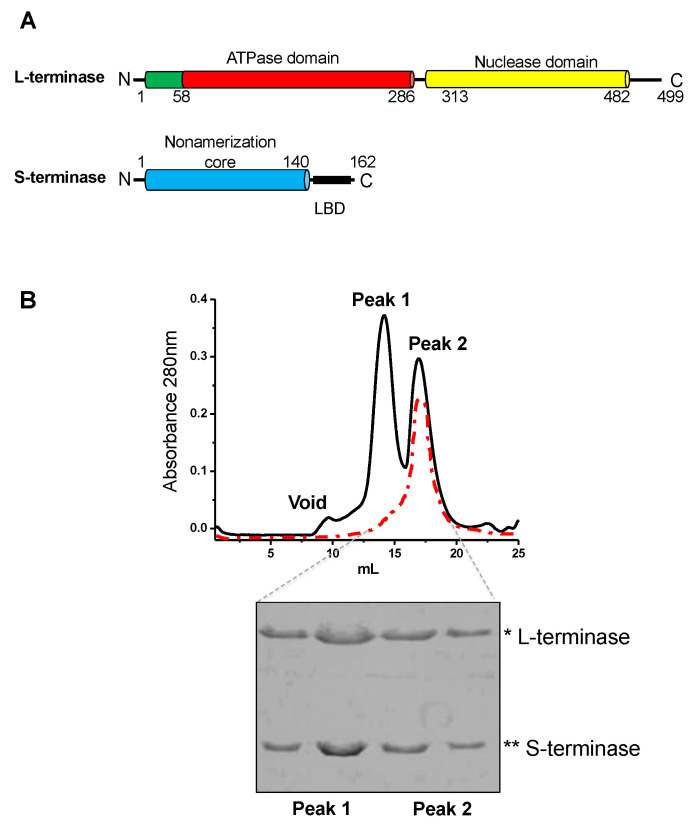
Co-expression and purification of the gp3:gp2 complex. (**A**) Domain organization of P22 S- and L-terminase subunits. (**B**) MBP-tagged gp3 is co-expressed with gp2 in *E. coli*. The complex is isolated on amylose beads, MBP tag is cleaved and the complex subsequently purified by size exclusion chromatography using a Superdex 200 column, which yields two peaks. Peak 2 is consistent with ~300–350 kDa, whereas peak 1 is larger (1 MDa–700 kDa). Peaks 1 and 2 are indistinguishable in SDS gel analysis and are initially in dynamic equilibrium. Peak 2 was run three times over gel filtration to obtain a single peak (red trace). Peak 2 was used for all studies herein. Two fractions from each peak were analyzed on SDS-Page gels. Large terminase (L-terminase) and small terminase (S-terminase) molecular weights are denoted by * and **, respectively.

To determine the structure and organization of the gp3:gp2 complex, Peak-2 (Figure 2) was analyzed with negative stain EM. To enhance the contrast of the sample, approximately 2–5 nm carbon was floated off mica support film and placed over C-flat grids containing 3 × 3 hole patterns with a 2 mm hole diameter and 0.5 μm hole spacing (Protochips (EMS) CF-2/0.5-4C-50, Morrisville, NC, USA). The grids were then glow discharged. Upon screening the appropriate sample concentration, 3 μL sample was applied to the grid for 1 min. The grid was gently blotted and passed through four 50 μL volumes of 2% uranyl formate. Subsequently, the grid was blotted, air-dried, and stored under desiccation.

#### 2.1.2. EM Data Collection and Processing

Because of sample heterogeneity, it was necessary to use the Random Conical Tilt (RCT) data collection strategy to obtain initial 3D models [34]. Image tilt pairs, untilted and −55°, were acquired for 1100 images using a Tecnai 12 electron microscope operating at 120 keV, with a dose near 20 e^−^/Å^2^ (Figure S1) sourced from a Denka M-3 LaB Phillips Cathode (product No. 1451) (Denka Company Ltd., Tokyo, Japan). The C-Flat grid 3 × 3 hole patterns were targeted for RCT using Leginon (NYSBC, New York, NY, USA), version 3.1 [35]. We applied a focus node at the center of the 3 × 3 holes, and subsequently captured images of the remaining 8 holes. Data processing was conducted using the APPION suite (NYSBC, New York, NY, USA), version 3.1 [36]. DogPicker (NYSBC, New York, NY, USA), version 0.2.1 was used to pick particles from all images [37]. Particles within image pairs were correlated using TiltAutoAligner (NYSBC, New York, NY, USA) [38]. Upon making stacks of the zero-tilt image particles (22,744) and corresponding tilted image particles, the zero-tilt images were iteratively classified using the CL2D algorithm [39]. This resulted in 18,767 zero-tilt particles. Class averages of zero-tilt particles matched the negative tilt particle reconstruction.

#### 2.1.3. Focused Classification and 2D Alignment

**Figure 3:**
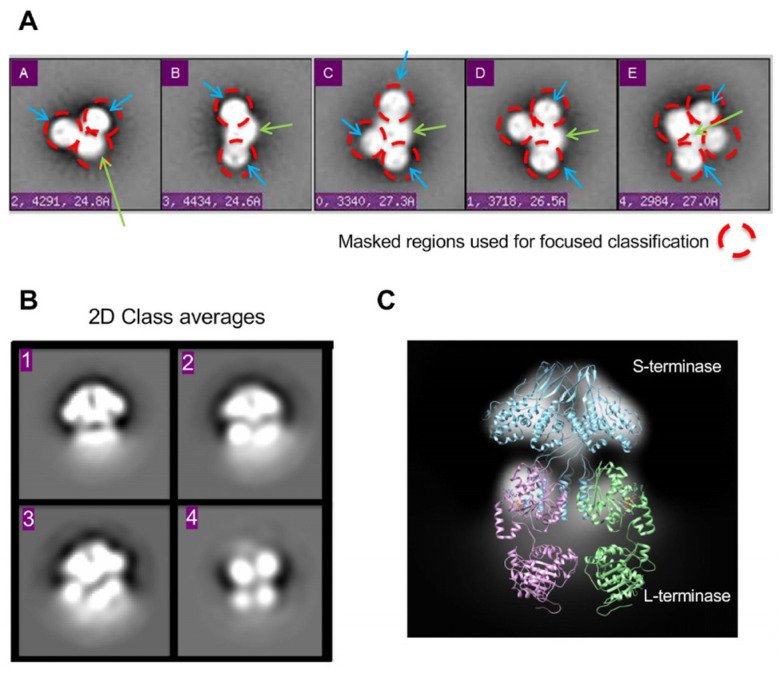
Focused classification in 2D. (**A**) Class averages representing various assembly states after reference-based alignment with SPIDER [40]. Masked regions for focused classification are circled in red. Putative gp3 and gp2 regions are denoted with blue and green arrows, respectively. Ellipsoid gp2 region denoted with a green arrow. (**B**) Resultant 2D class averages with RELION after masking. (**C**) The 9-mer S-terminase density strongly resembles the P22 gp3 crystal structure with C-terminal helices modeled (PDB 3P9A), giving confidence in focused classification results. The 2D density shows S-terminase (blue) with two gp2s (purple and green) attached at S-terminase C-terminus. The full-length gp2 homolog is from Sf6 (PDB 4IEE) [25].

After classification with CL2D produced class averages where side views of gp3 were discernable, representative class averages were used for referenced-based alignment using SPIDER (System for Processing Image Data from Electron microscopy and Related fields) [40]. Following referenced-based alignment, a mask was generated to select each lobe from an image of each class average (Figure 3A). A low-pass Gaussian filter of 0.0125 was applied to the mask. The mask was then used to crop out corresponding areas for each aligned class average. All of the masked particles from each class average were combined into stacks with the class they originated from, centered, and subsequently submitted to 2D classification in both RELION (REgularized LIkelihood OptimizatioN) [41] and SPARX (Single Particle Analysis for Resolution eXtension) [42], separately.

#### 2.1.4. Two-Dimensional Alignment

Unmasked images were aligned and clustered iteratively using ISAC (Iterative Stable Alignment and Clustering) [43]. Irreproducible clusters were used as a seed for subsequent rounds of alignment and clustering for up to twelve generations. ISAC produced the best class averages for this heterogenous dataset.

#### 2.1.5. Native MALDI Mass Spectrometry

Prior to native mass spec measurement, the purified S:L-terminase complex was buffer-exchanged into 150 mM aqueous ammonium acetate (AmAc) (pH 8.0), by ultrafiltration (Vivaspin 500, Sartorius Stedim Biotech, Göttingen, Germany) with a 10 kDa cutoff. A volume of 1–2 µL of sample, at a final concentration of 2 µM, was loaded into a nanoflow gold-plated borosilicate electrospray capillary (made in-house). The higher-order oligomers of S:L-terminase complex were analyzed on a modified QTOF-2 (Waters/MS Visions) operated on positive ion mode. Xenon was used as collision gas. MS parameters: backing pressure, 10 mbar; capillary, 1300 V; cone, 60 V; extracted cone, 0 V; pressure in the collision cell, 2 × 10^−2^ mbar; collision energy, 30 V. The sample was analyzed on a modified Exactive Plus EMR Orbitrap instrument (Thermo Fisher Scientific, Bremen, Germany) over an *m*/*z* range of 500–20,000 [44,45]. Manual tuning of the voltage offset on the flatapole, transport multipole, and ion lenses was used for mass filtering of the incoming protein ions, as previously described [46]. Nitrogen was used for the HCD cell at a gas pressure of 6–8 × 10^−10^ bar. Mass spec parameters: spray voltage, 1.3–1.4 V; source fragmentation, 30 V; source temperature, 250 °C; collision energy, 40–50 V; 10,000 resolution at *m*/*z* 200. Maxent was used to deconvolute the peaks and determine molecular weights.

### 2.2. Procapsid Production and Purification

P22 phage and *Salmonella* were provided by Peter Prevelige (University of Alabama at Birmingham). The *Salmonella* strains used were derivatives of *Salmonella* typhimurium LT2, DB700 suppressor minus (leuA^−^ 414(am) sup^o^) and supE derivative suppressor plus MS1363. The supplied phage contains an amber mutation in gp2 and gp3. This phage was used to infect MS1363 suppressor plus *Salmonella*, which leads to intact phage and enables high titer production. Upon reaching the desired titer of 10^2^ phage/mL, DB7000 suppressor minus *Salmonella* was infected with a multiplicity of infection (MOI) of 7. This enabled production of procapsids without packaged double-stranded DNA. Cell pellets were lysed and clarified before loading onto a linear 10–40% sucrose gradient. Procapsids were extracted from the side of the tube using an 18-gauge needle and subsequently loaded onto a CsCl step gradient where they migrate to the d = 1.2/1.4 CsCl interface (Figure S2), as previously described [47].

### 2.3. Native Agarose Gel Electrophoresis

Native agarose gel electrophoresis was conducted as previously published [47]. Briefly, native agarose gels were prepared in Tris-borate-magnesium (TBM) buffer (45 mM Tris, 45 mM borate, 1 mM MgCl_2_). Reaction conditions utilized 0.5 μg procapsid and 62 μg terminase complex on ice for 30 min in the presence of 0.01% or 0.1% glutaraldehyde. Crosslinking was quenched using 1 M Tris-HCl pH 7.0. The samples were then mixed with 4× loading buffer (1.5 × TBM, 12.5% *v*/*v*) Ficoll, 0.01% (*w*/*v*) bromophenol blue, 0.02% NaN_3_), and electrophoresed at 4 V/cm for three hours at 4 °C. After electrophoresis, the gel was gently rocked in 50 mM EDTA for 20 to 30 min. The gel was then stained for one to two hours with Coomassie blue (10% (*v*/*v*) methanol, 10% (*v*/*v*) acetic acid, 0.01% (*w*/*v*) Coomassie blue R-250) prior to dehydration with a gel drier. The gel was destained overnight in 10% acetic acid.

## 3. Results

To examine the structure and stoichiometry of the gp2-gp3 complex, peak 2 from gel filtration (Figure 2) was visualized by negative stain EM. Peaks 1 and 2 are indistinguishable in the SDS gel analysis and in the TEM 2D classification. Initially, the peaks are in dynamic equilibrium. Iterative gel filtration of peak 2 yields a single peak (the red trace in Figure 2B). For this reason, peak 2 was used for all of the EM and native mass spectroscopy work. Particles totaling 35,534 were picked from 1101 images using the Difference of Gaussians (DoG) Picker. These particles were initially sorted to remove poor particles using reference-free techniques, resulting in 18,767 particles. Subsequently, representative class averages were used as templates for reference-based alignment to separate the different assembly and conformational states.

### 3.1. Multiple (gp3)_9_ in gp2-gp3 Complex

The individual gp2 and gp3 components identified in the class averages were based on first recognizing gp3 (Figure 3A). Gp3 was easily recognized with striking side views of its nonamer β-hairpin and α-helical core based on projections of the crystal and EM structures [2]. The centralized ellipsoid density was featureless but was assumed to be gp2 (Figure 3A). The poor resolution of the gp2 region could be explained by the flexibility of the gp2 domains. At this early stage, the class averages contained only one, two, or three (gp3)_9_ assemblies.

### 3.2. Focused Classification Reveals gp2-gp3 Domain Interaction

To obtain domain-level resolution in the putative gp2 ellipsoid density region, a focused classification strategy was utilized. Briefly, particles were aligned to major class averages consisting of two or three (gp3)_9_ subunits bound to the gp2 ellipsoid density (Figure 3A). Individual stacks were made for each type of (gp3)_9_ organization, and the particles were again aligned to corresponding 2D class averages using SPIDER [40]. After verifying via a visual analysis that the particles were properly aligned, a mask was drawn around the gp3 density and a small portion of the ellipsoid density for each class average. The mask was then used to crop the density of the aligned particles using the corresponding class average. The cropped particles containing gp3 and a small portion of ellipsoid density were next subjected to 2D classification using RELION [41]. This focused 2D classification strategy produced a class average where gp2 density interacting with gp3 could be resolved. Beneath the gp3 mushroom-like density, two spherical densities could be resolved (Figure 3B). Each density is the size of an individual gp2 domain and agrees with the (gp3)_9_:2(gp2) terminase holoenzyme reconstruction previously published [30]. These results support that the ellipsoid density with multiple gp3 subunits attached corresponds to a gp2 core (Figure 3C).

### 3.3. Variable (gp3)_9_ Subunits Attach to Large Terminase Core

**Figure 4:**
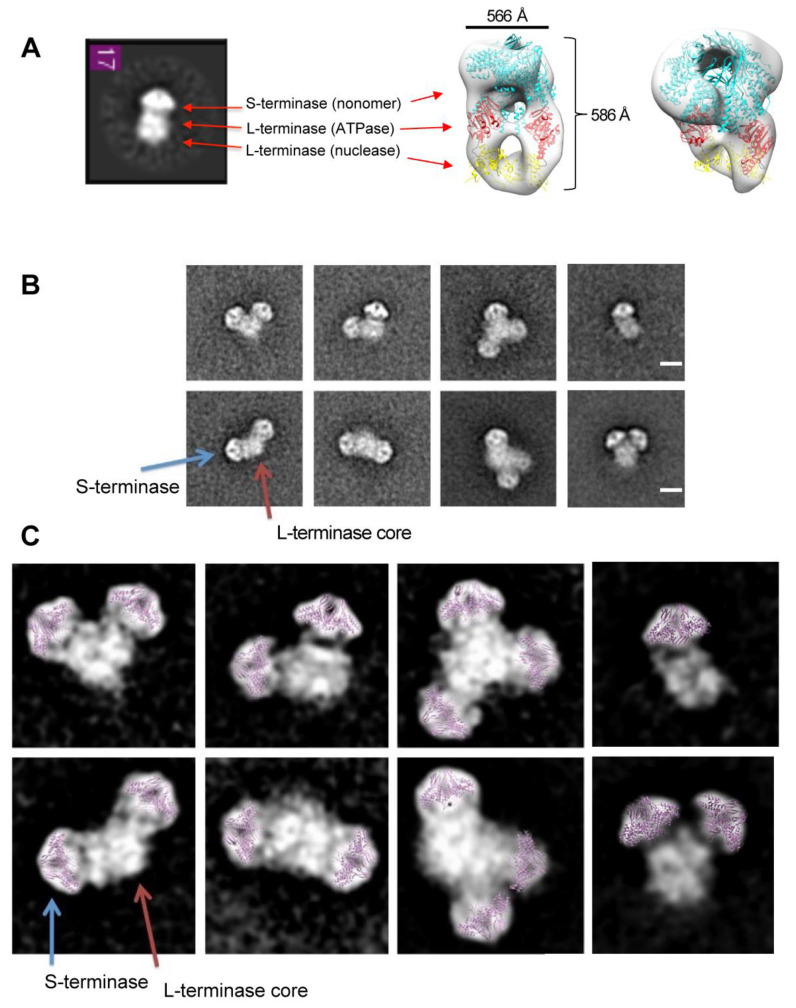
S-terminase (gp3)_9_ attached to an L-terminase (gp2) core. (**A**) Class average matches P22 terminase holoenzyme complex containing 1 (gp3)_9_ (cyan) subunit and 2 gp2 (ATPase-red and nuclease yellow) subunits (EMD-6429). (**B**) Iterative Stable Alignment and Classification (ISAC) 2D class averages. Class averages contained one, two, or three (gp3)_9_ subunits (blue arrow) attached to a gp2 core (red arrow). Scale bar is 100 Å. (**C**) Class averages are from (**B**). Gp3 residues 1–139 (purple) manually docked into representative gp3 density.

All the heterogeneity in the class averages observed was the result of dynamic equilibrium. The initial class averages were low in resolution in the (gp3)_9_ region because the programs could not manage alignment with concurrent conformational heterogeneity and variable stoichiometry in the dataset, likely because they focus on aligning particles to their overall shape. To obtain better detail and define gp3 localization in the heterogeneous terminase holoenzyme populations, the 2D classification of entire raw particles was performed using the ISAC algorithm. ISAC clearly showed holoenzyme complexes consisting of one (Figure 4A), two or three gp3s with different relative positions (Figure 4B). The (gp3)_9_ subunit predominantly appeared with a side view-preferred orientation on the grid (Figure 4B,C).

**Figure 5:**
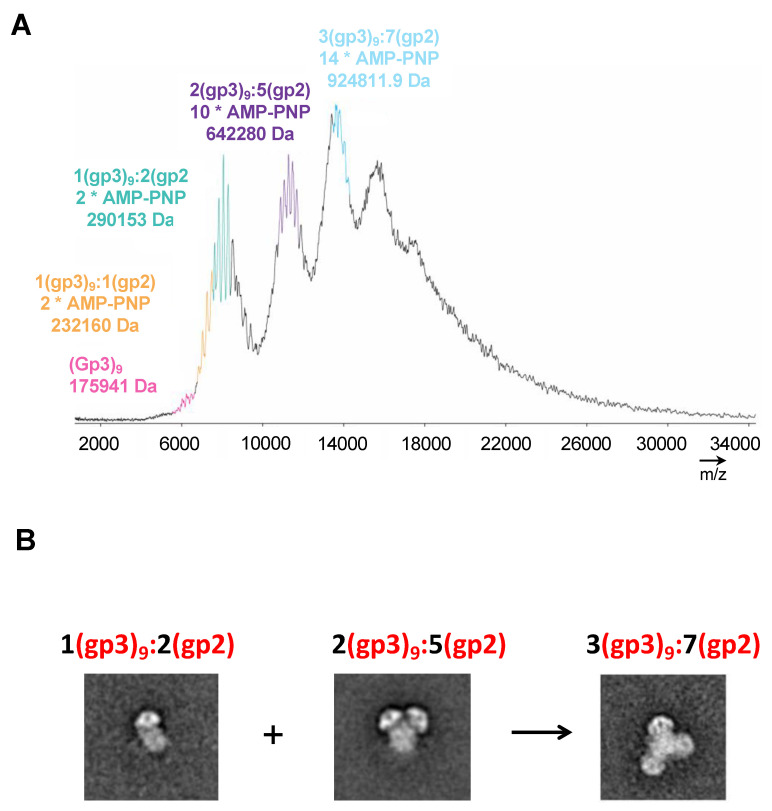
Mass spectrometric analysis of purified gp3:gp2 complex. (**A**) The differential assembly is consistent with at least two distinct gp3:gp2 populations of ~290 kDa (stoichiometry (1 × gp3) + (2 × gp2:AMP-PNP)) and ~642 kDa (stoichiometry (2 × gp3) + (5 × gp2:AMP-PNP)) and a ~925 kDa oligomer of the first two complexes combined (stoichiometry (3 × gp3) + (7 × gp2:AMP-PNP)). (**B**) Representative class averages of the three major assemblies identified by mass spec.

To resolve the number of gp2 subunits associated with each terminase holoenzyme assembly, a native matrix-assisted laser desorption/ionization (MALDI) mass spectrometric analysis of the purified samples was performed on peak 2 (the red trace in Figure 2B). The analysis revealed mass measurements consistent with 1(gp3)_9_:1(gp2) *(2AMP-PNP), 1(gp3)_9_:2(gp2) *(2AMP-PNP), 2(gp3)_9_:5(gp2) *(10AMP-PNP), and 3(gp3)_9_:7(gp2) *(14AMP-PNP) (Figure 5A). There are at least two populations for the holoenzyme complex. One population is approximately 290 kDa consisting of 1(gp3)_9_:2(gp2-AMP-PNP). A second peak is around 642 kDa consisting of 2(gp3)_9_:5(gp2-AMP-PNP). A third peak consisting of 3(gp3)_9_:7(gp2-AMP-PNP) is likely formed by a combination of peaks 1 and 2. The mass spectroscopy results support the main populations initially identified with transmission electron microscopy (Figure 5B).

### 3.4. Binding of Terminase Complex to P22 Procapsid

**Figure 6:**
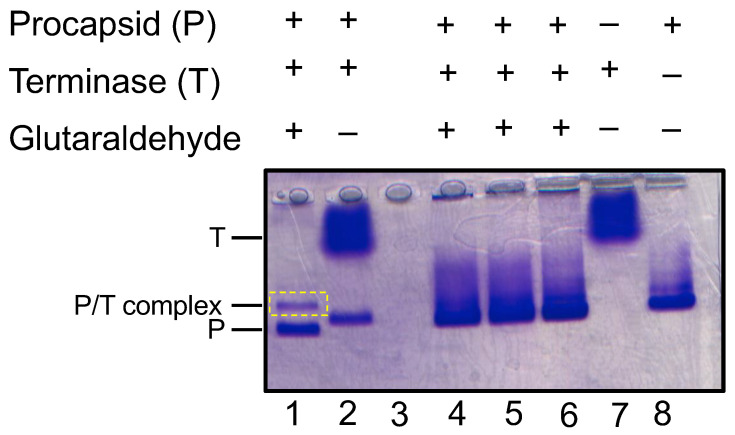
Generation of procapsid–terminase complex. Native agarose gel with procapsid (P) incubated with terminase complex (T) forms a new complex (P/T) in the presence of glutaraldehyde crosslinking. Lane 1 contains 0.01% glutaraldehyde and lanes 4–6 contain 0.1% glutaraldehyde and are replicates of the same experiment. Terminase and procapsid alone are shown in lanes 7 and 8, respectively. The crosslinked procapsid–terminase complex appears in lane 1 denoted as P/T complex (yellow dashed rectangle).

To examine if the terminase complex sample discussed herein could interact with the P22 portal protein in procapsids, procapsids were incubated with the terminase complex and analyzed using native agarose electrophoresis. In this native gel, which separates by surface charge to mass, the terminase migrated slower than the procapsid (Figure 6). The incubation of the procapsid and terminase only produced a new species in the presence of glutaraldehyde (Figure 6, Figures S3 and S4). In fact, a discrete procapsid–terminase complex band appeared with 0.01% glutaraldehyde, while incubation with 0.1% glutaraldehyde produced a smear.

## 4. Discussion

DsDNA bacteriophages assemble to form a procapsid to which an L-/S-terminase complex binds and brings concatemeric dsDNA and packages via a headful packaging mechanism. The L-/S-terminase complex is transient and dissociates after packaging is complete followed by binding of the tail machinery proteins. Recent studies have shown that purified portal protein can associate with L-terminase via a pulldown assay [48]. Moreover, in other phages, L-terminase has been found to assemble into a pentamer when associating with the head of the virus [27,28,49,50]. Herein, we demonstrate that bacteriophage P22 L-terminase can also assemble five gp2s with two S-terminase 9-mers attached. The purified complex yields homogenous bands on an SDS gel for L- and S-terminase. However, the native complex was in dynamic equilibrium with multiple assembly states. Iterative gel filtration yielded a homogenous peak, which still contained three main complexes verified by TEM and native mass spectroscopy. We propose that the class averages shown herein that represent the pentameric L-terminase bound to two S-terminase 9-mers is the physiological complex that facilitates genome packaging for bacteriophage P22.

Terminases are transient complexes that package viral genomes into preformed procapsids. After nearly a decade since the first moderate-resolution P22 terminase holoenzyme was determined, a high-resolution cryo-EM structure is still lacking. This is likely due to the biological heterogeneity observed with this complex illustrated herein. Unlike bacteriophages whose terminase complex is made of two proteins (L-terminase and S-terminase), herpesviruses have three proteins that comprise the terminase complex (pUL15, pUL28, and pUL33). Recently the terminase complex for herpesvirus has been determined and expressed as monomeric, hexameric, or dodecameric in form [51]. Thus, the heterogeneity observed for herpesvirus terminases, which are expressed in three main stoichiometries, are consistent with that observed herein for the P22 terminase complexes.

We found that the P22 terminase complex can assemble into three main classes: 1(gp3)_9_:2(gp2), 2(gp3)_9_:5(gp2), and 3(gp3)_9_:7(gp2). The variability in subunit assembly is supported by native mass spectroscopy, indicating that the observed assemblies are not an artifact of negative stain sample preparation. Additional experiments to validate the genome-packaging functionality of the complexes shown herein are warranted. Our data illustrate structural insights with in vitro observations. We note that DNA binding or packaging by terminases in pac systems like P22 is often weaker in vitro compared to cos systems, which may influence the detection and stability of such assemblies in solution [52]. While the complexes herein show features consistent with biological functions (e.g., procapsid binding and AMP-PNP binding), further experiments would be required to directly demonstrate DNA packaging activity. We propose the differential assembly of these complexes is due to S-terminase C-terminal helices that are free to associate with additional L-terminase ATPase subunits. The subsequent binding of an additional S-terminase nonamer enables additional L-terminase subunits to bind. The C-terminal helices of S-terminase fit the canonical definition of an intrinsically disordered region (IDR) according to PONDR (Molecular Kinetics, Inc., Indianapolis, IN, USA), an IDP software (Figure S5). Moreover, L-terminase also contains several IDRs that could contribute to the conformational and structural heterogeneity observed herein. The intrinsic disorder distribution within the protein sequence of small and large terminases is similar across species (Figure S5). The P22 terminase and different stoichiometries of HSV terminase both have three main assemblies. There are three main assemblies observed in HSV [51] and there are three main assemblies in P22 shown herein. We show similar regions of disorder for both of these viruses (Figure S5). We propose this variability in disorder accounts for the variability in the stoichiometry. Previous structural studies show that L-terminase is monomeric in solution but forms a pentamer at the portal vertex [27,28], similar to the phi29 DNA packaging motor [52]. Based on this, it is likely that the holoenzyme with 2(gp3)_9_:5(gp2) can directly bind and headful package the concatemeric genomic DNA inside the P22 procapsids. Our data show that one or more of the complexes are relevant because they bind procapsids in the presence of gentle glutaraldehyde crosslinking.

A nearly complete structural characterization exists for bacteriophage P22, ranging from mature viral assembly [14,53] to genomic injection conduit formation [54], with most protein structures and their locations known for the virus. There are models of terminases interacting with DNA that have been proposed for genome packaging [55,56]. Ultimately, determining the structure and mechanism of terminases interacting with procapsids [57] will shed light on which model is correct. Future experiments for the cryo-EM structure of the P22 terminase complex bound to procapsids may be facilitated by supplementing with P22 genomic DNA or gentle crosslinking, as suggested herein [58].

## Data Availability

All relevant data are contained within this publication.

## Supplementary Materials

The following supporting information can be downloaded at https://www.mdpi.com/article/10.3390/pathogens13121066/s1, Figure S1: Random Conical Tilt data acquisition; Figure S2: Random Conical Tilt data acquisition; Figure S3: P22 Terminase Complex; Figure S4: Isolated Terminase and Procapsid lack a crosslinked product; Figure S5: Intrinsic disorder region (IDR) distribution for P22, Sf6, and HSV-1 terminases.
