# Supplementary material for: Variable Assembly and Procapsid Binding of Bacteriophage P22 Terminase Subunits in Solution
Source: Pathogens. 2024 Dec 3;13(12):1066. doi: 10.3390/pathogens13121066 (PMC11728703; doi:10.3390/pathogens13121066)
Supplement: Supplementary file 1 [file pathogens-13-01066-s001.zip › pathogens-3281609-supplementary.pdf]

# Supplementary Materials

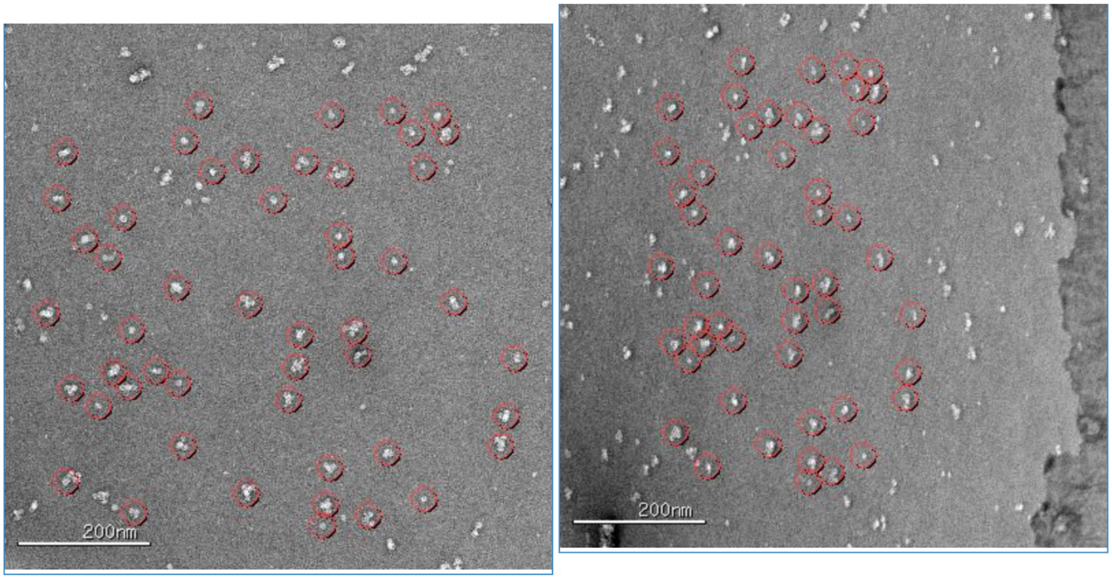


**Figure S1. Random Conical Tilt data acquisition.** To improve sample contrast, sample was applied to thin carbon layer over the holes of C-Flat grids. Both zero (left) and -55º (right) image pairs were collected with a nominal magnification of 52,000×, 1mm defocus, 2.05 Å pixel size and a dose of 20.15 $e^{-}/Å^{2}$. Particles were picked (red circles) using Difference of Gaussian algorithm.

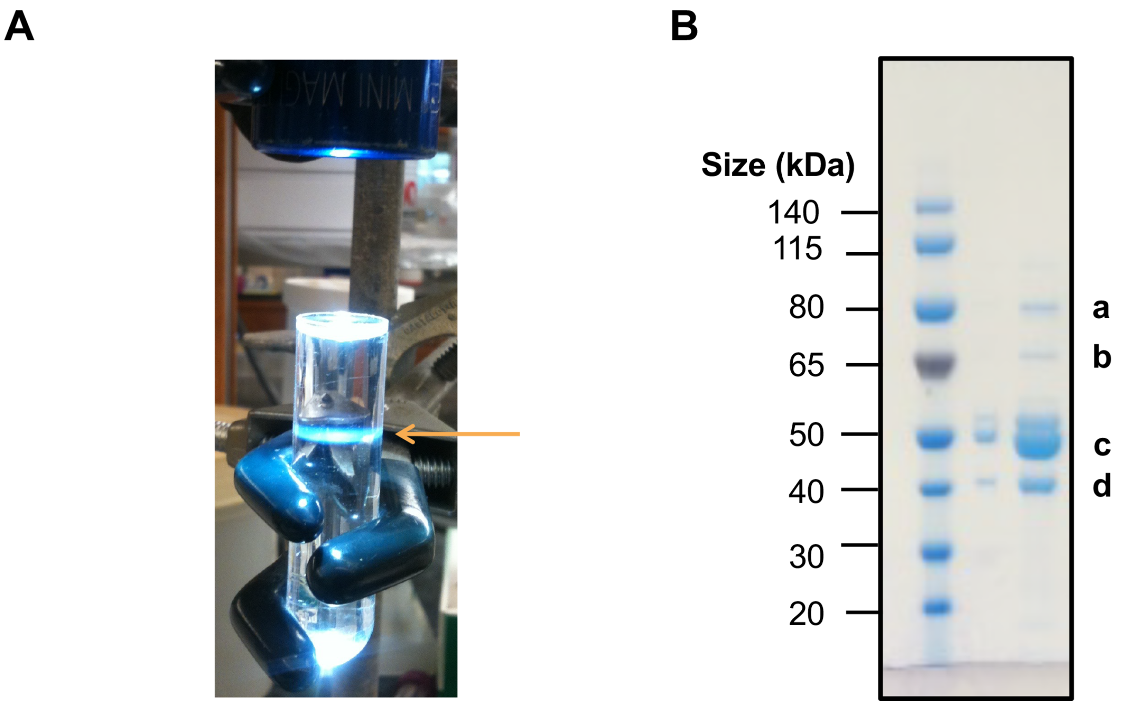


**Figure S2. Random Conical Tilt data acquisition.** To improve sample contrast, sample was applied to thin carbon layer over the holes of C-Flat grids. Both zero (left) and -55º (right) image pairs were collected with a nominal magnification of 52000x, 1mm defocus, 2.05 Å pixel size and a dose of 20.15 $e^{-}/Å^{2}$. Particles were picked (red circles) using Difference of Gaussian algorithm.

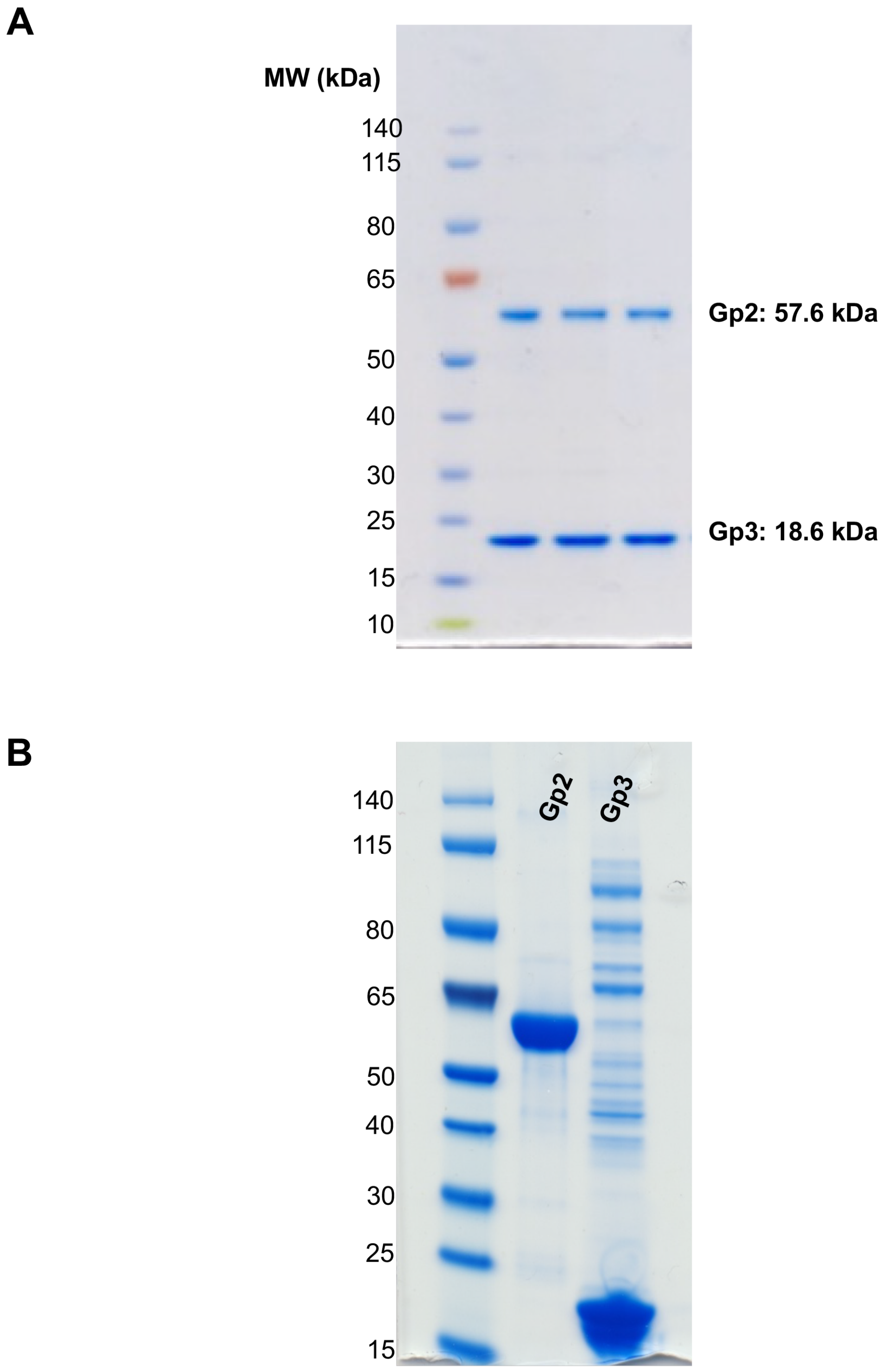


**Figure S3. P22 Terminase Complex.** (**A**) SDS-PAGE Coomassie gel showing triplicate runs of the gp3:gp2 complex. (**B**) Coomassie-stained SDS-PAGE gel depicting isolated gp2 and gp3 proteins.

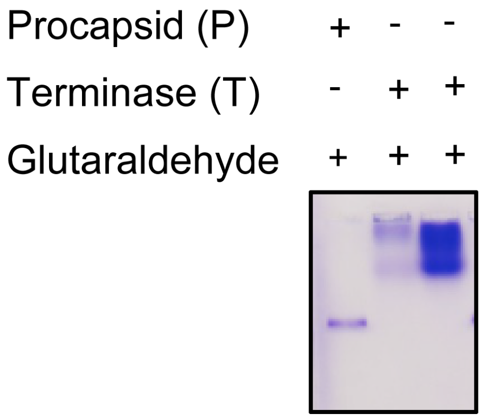


**Figure S4.** **Isolated Terminase and Procapsid lack a crosslinked product**. Native agarose gel with procapsid (P) or 2 different concentrations of terminase (T) incubated in the presence of glutaraldehyde crosslinking. All lanes contain 0.01% glutaraldehyde.

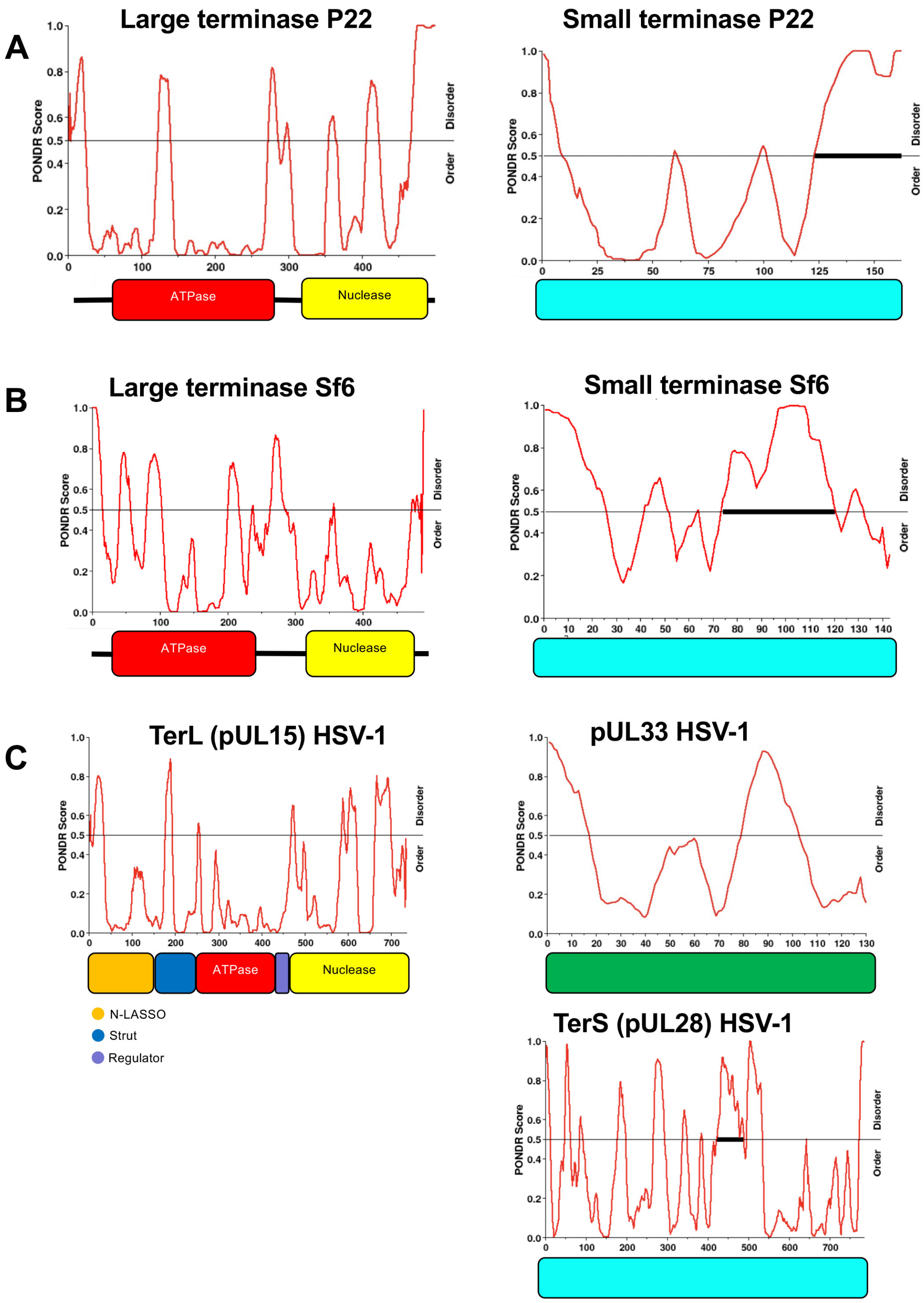


**Figure S5. Intrinsic disorder region (IDR) distribution for P22, Sf6, and HSV-1 terminases.** PONDR analysis indicates similar IDR's for L-terminases (left) across different species. Similar IDR's are also observed for small terminases (right). The ratio of order to disorder is shown for: (A) P22– gp2 and gp3; (B) Sf6– gp2 and gp1; (C) HSV-1– pUL15 (TerL), pUL33, and pUL28 (TerS).
